# Dedifferentiation of esophageal progenitors in metaplasia and cancer

**DOI:** 10.1080/23723556.2021.1991758

**Published:** 2021-10-21

**Authors:** Alizée Vercauteren Drubbel, Benjamin Beck

**Affiliations:** a Faculty of Medicine, Erasme Campus of Université Libre de Bruxelles (ULB), Institute of Interdisciplinary Research (IRIBHM), 808 Route de Lennik, Brussels, 1070, Belgium; b Welbio/FNRS Principal Investigator, Iribhm, ULB/Faculty of Medicine, Erasme Campus of Université Libre de Bruxelles (ULB), 808 Route de Lennik, Brussels, 1070, Belgium

**Keywords:** Cell plasticity, dedifferentiation, transdifferentiation, metaplasia, cancer

## Abstract

Barrett syndrome is a squamo-columnar metaplasia increasing the risk of developing esophageal adenocarcinoma. Recently, we showed that esophageal cells can transdifferentiate into undifferentiated columnar cells *in vivo*. Here, we discuss about the potential of these cells to be a reservoir for intestinal metaplasia and/or esophageal adenocarcinoma.

## Author’s view

Barrett esophagus (BE) is a premalignant condition that increases by 50 times the risk of developing esophageal adenocarcinoma (EAdC). This metaplastic condition is associated with gastro-esophageal reflux and comprised a mosaic of gastric and intestinal cell types. In many countries, intestinal metaplasia characterized by goblet cells and the expression of caudal type homeobox 2 (CDX2) is considered at higher risk of progressing to cancer.^1^ The processes leading to the development of such metaplasia are complex. Notably, it is still unclear whether BE involves the replacement of the squamous epithelium by another cell population, the transdifferentiation of esophageal cells, or both mechanisms.

Many *in vitro* studies sustain transdifferentiation, but a complete conversion of esophageal keratinocytes into intestinal cells has never been reported *in vivo*.^2^ Conversely, *in vivo* studies showed that the squamous epithelium can be replaced by columnar cells from the squamo-columnar junction (SCJ).^2^ This transition epithelium resembles the columnar epithelium lining the foregut during embryogenesis.^3^ Although it has not been demonstrated, such an undifferentiated status may facilitate the activation of an intestinal differentiation program. In a mouse model of gastro-esophago-jejunostomy that mimics gastro-esophageal reflux disease, some intestinal metaplasia are surrounded by squamous tissue, suggesting they may arise from keratinocytes.^4^ In line with this, an elegant study showed that a subset of keratinocytes from the SCJ can transdifferentiate upon ectopic expression of the human transcription factor (TF) CDX2, while esophageal progenitors cannot.^5^ These results demonstrate that BE may have multiple cellular origins and that keratinocytes from the SCJ have unique properties.

**Figure 1.**
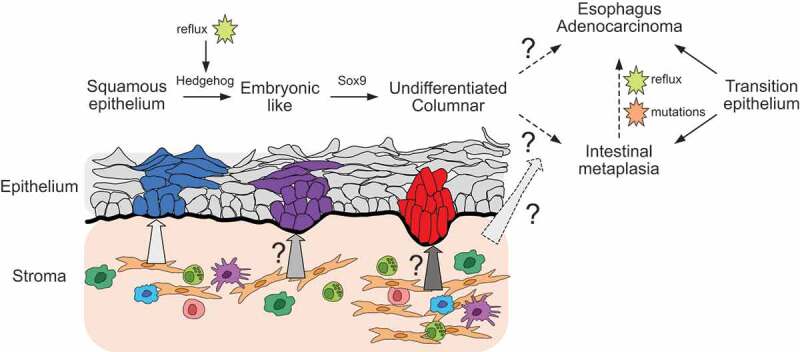
*Potential contribution of keratinocytes to esophageal metaplasia and adenocarcinoma*. Upon activation of the Hedgehog pathway, esophageal cells dedifferentiate into embryonic-like cells and then a subset of these cells undergoes a squamous-to-columnar conversion in a SRY-box Transcription Factor 9 (Sox9) dependent manner. These *de novo* undifferentiated columnar cells may constitute a reservoir for some intestinal metaplasia and/or adenocarcinoma. Dashed arrows represent the hypothetical evolution of these columnar cells through interactions with the microenvironment and/or other factors such as reflux or mutations. It has been already demonstrated that undifferentiated columnar cells from the transition epithelium at the squamo-columnar junction can initiate intestinal metaplasia and/or adenocarcinoma upon chronic gastroesophageal acid reflux and/or driver mutations

Our group recently showed that the hedgehog pathway (HH) is more active in keratinocytes at the SCJ than in the esophagus.^6^ Interestingly, this pathway is activated in the esophagus epithelium upon chronic gastro-esophageal reflux. We showed that the activation of the HH pathway in esophagus epithelium triggers an embryonic-like transcriptomic and epigenetic program leading to the dedifferentiation and then the columnar conversion of a subset of esophageal progenitors. Although several selected intestinal markers were upregulated upon HH-induced columnar conversion, no sign of goblet cells nor Cdx2 expression was observed in this model. Other elements might thus be necessary to turn on an intestinal differentiation program. The model of HH-induced columnar conversion will thus allow determining whether undifferentiated columnar cells have the competence to initiate intestinal metaplasia and if this step is required for tumorigenesis (Figure 1).

Our data show that HH-induced columnar conversion depends on SRY (sex determining region Y)-box 9 (Sox9). Interestingly, Sox9 promoter contains putative binding sites for Smad TF. Hence, squamous-to-columnar conversion may be regulated by cues from the microenvironment, notably signaling activating Smad, such as BMP (Bone morphogenetic protein) and TGFb (Transforming growth factor beta).^6^ In mouse models of BE, the accumulation of inflammatory cells and other stromal cell types due to chronic reflux contribute to the activation of different signaling pathways via secretion of cytokines and chemokines.^2^ Extrinsic factors such as inflammation, reflux and obesity also modulate progression of intestinal metaplasia toward malignancy. Hence, modifying cues from the environment in the model of HH-induced esophageal cell reprogramming could help identifying the role of extrinsic factors in the initiation of intestinal metaplasia and/or EAdC (Figure 1).

It is believed that the transformation from normal esophagus to metaplasia and then EAdC is driven by a stepwise accumulation of mutations.^7^ Interestingly, although EAdC is associated to columnar metaplasia, about 50% of EAdC patients do not have evidence of intestinal metaplasia at the time of diagnosis. Using a multi-omic profiling of freshly isolated human cells, the group of *Rebecca Fitzgerald* recently suggested that BE and EAdC would arise from undifferentiated columnar cells.^8^ Moreover, according to this study, intestinal differentiation would not be necessary for cancer initiation. Hence, *de novo* undifferentiated columnar cells in the esophagus may constitute a reservoir for EAdC initiation, even if they fail to achieve an intestinal differentiation program. The model of HH-induced squamous-to-columnar conversion may be useful to address this question since it allows to generate undifferentiated columnar cells in the esophagus. This will require to activate specific oncogenic hits in HH-stimulated esophageal cells (Figure 1).

In mouse, the forestomach is lined with a squamous epithelium like the esophagus. It has been shown that SRY-box Transcription Factor 2 (Sox2), one of the most frequent gene amplified in esophagus squamous cell carcinoma, leads to the development of tumors in the forestomach but not in the esophagus, when overexpressed in mouse foregut epithelium.^9^ This difference relies at least partially on the level of inflammation and signal transducer and activator of transcription 3 (Stat3) expression, which is higher in the forestomach and promotes carcinogenesis.^9^ On one hand, this observation highlights the influence of the microenvironment on cancer initiation. On the other hand, the same phenotype could be observed in models of metaplasia and EAdC, and these histological lesions might appear preferentially in the forestomach. Finally, metaplasia and EAdC may also originate from submucosal glands, which are absent in rodent esophagus. Anatomical differences between mouse and human may therefore constitute a limitation in the study of BE and EAdC initiation from esophageal cells.

In conclusion, our recent data show that esophageal keratinocytes can transdifferentiate to achieve a full columnar conversion *in vivo*. However, it is unclear what would be the fate of such undifferentiated columnar cells in the esophagus. Further experiments will be important to determine whether these *de novo* columnar cells can initiate EAdC and if a preliminary step of intestinal differentiation is required.
